# A Woman’s Edition

**Published:** 1896-05

**Authors:** 


A Monthly Review of Medicine and Surgery.

*EDITORS:*

THOMAS LOTHROP, M. D. - - WM. WARREN POTTER, M. D.

All communications, whether of a literary or business nature, should be addressed to the managing editor: 284 Franklin Street, Buffalo, N. Y.

Vol. XXXV. MAY, 1896. No. 10.

### A WOMAN'S EDITION.

IT AFFORDS us much pleasure to announce to our subscribers and all interested in the welfare of the Journal that our next issue, June, 1896, will be distinctively and absolutely a woman's number. This means that the entire magazine will be the work of woman's brain and hand. The original communications, dealing with several important subjects, will be written by women; the department of progress in medical science will be selected, abstracted and prepared by women; the society proceedings will be reported by women, and the editorial department, including book reviews, personals, literary notes, miscellaneous and news items, all will be the work of women physicians who are eminently fitted for the duties they have kindly undertaken.

The arrangement, proof-reading and general editorial work, too, in all its details, will be carried on by women, so that, as we have before stated, the June issue of the magazine will distinctively represent woman's work in medical literature. We confidently predict that the number in contemplation, not only in the excellence of its material, but in its illustrations and its general make-up, will be one of the most attractive medical magazines ever issued.

It must be borne in mind that such an undertaking is no light task and deserves the most liberal support on all sides; hence we trust our subscribers will manifest an interest in this proposed woman's edition by ordering extra copies with liberality. They will be supplied at 25 cents apiece.

We are not at liberty to disclose the names of the enterprising women physicians who have banded themselves together in this useful and instructive effort, nor are we permitted to mention the table of contents that is preparing, for we are trying to demonstrate the masculine ability to keep a secret ; but the general scheme has been sufficiently unfolded to us to enable us to affirm that the first medical magazine ever conducted in Buffalo by women will be a lasting testimonial to the professional ability of those who are to contribute to its columns.

The idea of a woman's edition was first suggested by a staunch friend of the JOURNAL, who, we are sure, will be much surprised as well as gratified to learn through this announcement, for the first time, that his suggestion has taken material form and is likely to be successfully demonstrated.

